# Oxytocin changes in women with emergency cesarean section: Association with maternal blues by delivery mode

**DOI:** 10.1016/j.heliyon.2023.e15405

**Published:** 2023-04-18

**Authors:** Eri Shishido, Shigeko Horiuchi

**Affiliations:** Graduate School of Nursing Science, St. Luke's International University, Tokyo, Japan

**Keywords:** Oxytocin, Emergency cesarean section, Maternity blues, Postpartum fatigue

## Abstract

**Introduction:**

Women with emergency cesarean section (CS) have presumed effects of an unscheduled surgery on their salivary oxytocin (OXT) level and psychological state. This study aimed to measure changes in the salivary OXT levels of women with emergency CS and change in the OXT levels by delivery mode, and to investigate the association between changes in OXT levels and maternity blues.

**Methods:**

We used a longitudinal observational study. The eligibility criteria were primipara pregnant women who were planning to have vaginal delivery. The salivary OXT levels of women were measured at 36 weeks gestation, 38 weeks gestation, 1 day postpartum, and 5 days after childbirth. Maternity blues was diagnosed using the Maternity Blues Scale (13 items), ‘Fatigue after Childbirth’ was diagnosed using the Visual Analogue Scale (0–100), and the subjective symptoms of fatigue was diagnosed using the Jikaku-sho shirabe. The three groups (“Without EA”, “With EA”, and “Emergency CS”) were analyzed separately. The changes in the oxytocin levels of women with emergency CS at four time points were analyzed by using a repeated measure analysis of variance.

**Results:**

The mean OXT levels of women with emergency CS (n = 6) were significantly lower at 5 days after childbirth than at 36 weeks gestation, 38 weeks gestation, and 1 day postpartum. There was a significant middle correlation between changes in the mean maternity blues scores between 1 day and 5 days, and the mean changes in OXT levels from 38 weeks gestation to 5 days after childbirth.

**Conclusion:**

It could be assumed that women with emergency cesarean section may be affected psychologically by the unplanned method of delivery. In the present study, it was not possible to analyze this association because of the small sample size; however, it is possible to clarify predictors as the sample size accumulates in the future.

## 1 Introduction

Oxytocin (OXT) is commonly known as the hormone of love, trust, and bonding. In addition to its role in the formation of mother-infant bonding, OXT also induces uterine contraction and breast milk secretion. Running, sexual intercourse, breastfeeding, nipple stimulation, and massage during pregnancy also promote the secretion of endogenous OXT [[1], [2], [3]]. By contrast, OXT has been associated with aggressive behaviors, such as excluding outgroup members to defend or protect ingroup members. It has also been associated with increased anxiety and fear in mice where OXT appeared to reinforce positive and negative memories [[4], [5], [6]].

Oxytocin has also been determined to be involved in mental health, such as during postpartum depression and the maternity blues. There have been three previous studies showing the association between plasma oxytocin levels and postpartum depression using the Edinburgh Postnatal Depression Scale (EPDS) during pregnancy through to the postpartum period. Two of these studies confirmed this association [7,8], but one did not [9]. Besides postpartum depression, maternity blues which has an onset within 10 days postpartum, may also be associated with oxytocin levels. Maternity blues occur in approximately 20% of women and is naturally relieved; however, it affects women's mental health and their maternal bonding with their baby.

Previous studies have shown the relation between OXT dosage and prophylactic bleeding control [[10], [11], [12]]. However, no studies have yet examined their association, both EA and CS use spinal and EA, and women who have CS and those who have EA showed similar changes in the OXT levels after birth. In addition, the present study could also help clarify the trend of OXT levels in women who have had an emergency CS.

Although OXT levels in women with vaginal delivery have been investigated [13], no studies to date have found an association between OXT levels and maternity blues or postpartum fatigue in women with emergency CS. The incidence of maternity blues at 2 days after childbirth has been reported to be more frequent in women with CS than in women who undergo CS than in women who experience vaginal delivery [14]. Moreover, protective factors for maternity blues indicate natural birth. Women with emergency CS are assumed to suffer from the effects of an unscheduled surgery; for example, altered OXT levels and psychological state [15,16].

The aims of this preliminary study are to measure changes in the OXT levels of women with emergency CS and the amount of change in the OXT by the mode of delivery from late pregnancy to early postpartum, and to investigate the association between the amount of change in the salivary OXT levels and the change in maternity blues/fatigue scores from 1 day to 5 days after childbirth.

## 2 Methods

### 2.1 Design

This was a longitudinal observational study that used saliva sampling and questionnaires at four time points.

### 2.2 Participants

The eligibility criteria were primipara pregnant women who were planning to have vaginal delivery; who were from 20 to 40 years old; and who could read and write in Japanese. The exclusion criteria were *women with* endocrine disease, mental disorder, epilepsy, obstetric complication with pregnancy induced hypertension, gestational diabetes mellitus, imminent abortion, infection with HIV/HBV/HCV, alcohol addiction, smoking habit, illicit drug usage, and easy bleeding in the oral cavity.

### 2.3 Setting

The salivary OXT levels was measured at four time points: 36 weeks of gestation (baseline), 38 weeks of gestation, 1 day after childbirth, and 5 days after childbirth. The settings and procedures were similar to those in previous studies [13]. Pregnant women who met the eligible criteria were selected from the medical records. When eligible pregnant women at 34 weeks of gestation selected had their maternity check-up, the researcher verbally explained our research using the explanatory booklet that contained information about the provision of confidentiality and anonymity of their data. Women also received a refusal form at that time with an explanation that they could withdraw from the study any time without any disadvantage. After obtaining consent, we decided what the first experiment day (36 weeks of gestation) would be with the participant.

Based on the Salimetrics guidelines (2015) [17], the participants were asked not to drink alcohol or caffeine, and not to see a dentist before the day of the experiment They were also instructed to finish their meal, brush their teeth 1-h before the experiment, and also were asked not to use lipsticks and lip balm [18]. They were asked not to use mobile phones during experiments, as the calls could change their mood. A minimum of 2 ml of saliva was required for saliva analysis; pooling in the mouth for 1 min, salivating for 1 min, and resting for 30 s; this was repeated three times. The collected salivary samples were immediately stored in a freezer at −80 °C (Cryo Porter CS-80C; Scinics Corp., Tokyo, Japan).

After saliva collection, the participants were asked to answer a paper questionnaire at 36 weeks of gestation and twice after childbirth.

This study was conducted at a national hospital in Tokyo, from January 2019 to June 2019.

### 2.4 Measurements

#### 2.4.1 Detection of OXT levels

The salivary OXT levels was measured at four time points: 36 weeks of gestation (baseline), 38 weeks of gestation, 1 day after childbirth, and 5 days after childbirth. OXT levels was assayed in duplicates by enzyme-linked immunosorbent assay (ELISA; ENZO Life Sciences, NY, USA) following the protocol of Carter et al. (2007) [19].

After thawing the saliva, 500 KIU/μL aprotinin was added to the salivary samples to inhibit metabolic breakdown of the proteolytic degradation. The ELISA manual states that the intra-assay and inter-assay coefficients of variability are <13.3% and <20.9%, respectively (Product Manual OXT ELISA kit; http://static.enzolifesciences.com/fileadmin/files/manual/ADI-900-153A_insert.pdf). OXT levels were determined by duplicate assay using ELISA. These analyses were conducted by Professor Takuya Shuo in the School of Pharmacy at Hokuriku University in Japan. The other details for the method to measure OXT levels are shown in the previous study by Shishido et al. (2021) [13]. After a simultaneous incubation at 4 °C, the excess reagents were washed away and substrate was added. Second, the enzyme reaction was stopped and the yellow color generated was read on a microplate reader at 405 nm. The measured optical density was used to calculate the oxytocin levels.

#### 2.4.2 Basic data of participations

Eight information items were collected using a questionnaire: age, height, weight, BMI, dysmenorrhea, marital status, living with partner, and feeling about pregnancy.

#### 2.4.3 Maternal and neonatal outcomes

The following data were collected: mode of delivery (vaginal delivery, emergency CS), amount of OXT (unit), duration of labor (min), instrumental delivery, induction of labor, augmentation of labor, intrapartum hemorrhage (g), degree of perineal tear, skin contact (SSC), gender of baby, Apgar scores (1 min/5 min), birth weight, and NICU admission immediately after birth.

#### 2.4.4 Postpartum

The following data regarding the postpartum period were taken from the medical records: Time from childbirth to first walking and breastfeeding.

#### 2.4.5 Questionnaires: Data at 1 day and 5 days after childbirth

Maternity blues was evaluated using the Maternity Blues Scale (13 items), ‘Fatigue after Childbirth’ using the Visual Analogue Scale (VAS：0–100 mm), and subjective symptoms of fatigue using the Jikaku-sho shirabe. The subjective symptom scale was developed by the Working Group of Industrial Fatigue, which is part of the Japan Society for Occupational Health [20]. This scale consisted of 25 items and 5 elements (1. feeling of drowsiness, 2. feeling of instability, 3. feeling of uneasiness, 4. feeling of local pain or dullness, and 5. feeling of eyestrains). There is no cut-off, but the higher the score, the more extensive the fatigue.

### 2.5 Statistical analysis

The collected data were descriptively analyzed. To determine if the data were normally distributed, a histogram was used to illustrate the distribution. Because the data were not normally distributed, Spearman's rank correlation coefficient was used to analyze the correlation.

Three groups (“Without EA”, “With EA”, and “Emergency CS”) were analyzed separately. The baseline characteristics and outcomes for the participants were compared for the three groups using a one-way analysis of variance or the chi-square test.

The changes in salivary OXT levels were compared between the groups. Additionally, the changes in the salivary oxytocin levels during an emergency CS at four time points were analyzed by using a repeated measure analysis of variance. The OXT levels in women with emergency CS were analyzed in 6 participants as 1 participant had undetectable OXT levels.

Statistical analyses were performed using IBM SPSS Statistics (version 25.0; Static Base and Advanced Statistics, IBM Japan, Tokyo, Japan). All statistical tests were performed with a two-sided 5% level of significance.

### 2.6 Ethics

This study was approved by the Research Ethics Committee of St. Luke's international university, Tokyo, Japan (18-A065). It was conducted in accordance with the “Ethical Guidelines on Medical Research for Human beings.” If an adverse event occurred from the implementation of the study as well as health damage to the participants, the researcher and hospital would immediately respond to ensure that appropriate treatment and other necessary measures could be taken. Health insurance would cover the provided treatment.

## 3 Results

### 3.1 Characteristics of the participants

The demographic data of the participants are shown in Table 1. There was no significant difference in any of the data between the three groups (i.e., with EA, without EA, and emergency CS).

**Table 1:** Characteristics of the participants (N = 65).

	With epidural anesthesia (n = 29)	Without epidural anesthesia (n = 29)	Emergency cesarean section (n = 7)	F	*P*-value
***Demographic data***					
Age >35 (years)	13 (44.8%)	9 (31.0%)	1 (14.3%)		0.29
<34 (years)	19 (55.2%)	25 (69.0%)	6 (85.7%)		
Height (cm)	159.9 (SD4.73)	159.5 (SD5.15)	160.1 (SD5.52)	0.06	0.93
Weight (before pregnancy)	51.1 (SD4.93)	51.4 (SD5.17)	53.8 (SD12.8)	0.55	0.57
BMI	19.9 (SD1.78)	20.1 (SD1.84)	21.9 (SD4.92)	2.24	0.11
Dysmenorrhoea	2 (6.9%)	1 (3.4%)	0 (0%)		0.62
Marital status	29 (100%)	28 (96.6%)	7 (100%)		0.62
Living with partner	29 (100%)	27 (93.1%)	7 (100%)		0.48
Feeling about pregnancy					
Very happy	27 (93.2%)	20 (69.0%)	7 (100%)		0.06
Happy	1 (3.4%)	8 (57.6%)	0 (0%)		
Neither	1 (3.4%)	1 (3.4%)	0 (0%)		

*P*-value for One-way analysis of variance, or Chi-square test (comparing three groups).

### 3.2 Maternal and neonatal outcomes

The maternal and neonatal outcomes of the participants are shown in Table 2. There was no significant difference between the three groups except for bleeding (p < 0.01), administration of exogenous OXT (units) (p < 0.007), Apgar score at 1 min (<6 points) (p < 0.04), NICU (p < 0.01), and Skin to Skin Contact (p < 0.001).

**Table 2:** Maternal and neonatal Outcomes (N = 65).

	With epidural anesthesia (n = 29)	Without epidural anesthesia (n = 29)	Emergency cesarean section (n = 7)	*P*-value
***Maternal outcomes***				
Administration of exogenous oxytocin (units)	3.72 (SD 1.81)	2.48 (SD 1.45)	4.43 (SD2.63)	0.007
Perineal trauma				
Intact perineum	0 (0%)	2 (6.9%)		0.13
1st degree tear	1 (3.4%)	1 (3.4%)		
2nd degree tear	28 (96.6%)	23 (79.3%)		
3rd degree tear	0 (0%)	3 (10.3%)		
Bleeding (ml) > 500 （ml)	17 (58.6%)	13 (44.8%)	0 (0%)	0.01
<500 （ml)	12 (41.4%)	16 (55.2%)	7 (100%)	
***Neonatal outcomes***				
Weight (g)	3000 (SD231.2)	3167 (SD375.8)	3060(SD622.3)	0.20
Sex Boy	12 (41.4%)	13 (44.8%)	4 (57.1%)	0.51
Girl	17 (58.6%)	16 (55.2%)	3 (42.9%)	
AP1min (<6)	3 (10.3%)	1 (3.4%)	2 (28.6%)	0.04
AP5min (<6)	1 (3.4%)	0 (0%)	1 (14.3%)	0.058
NICU	0 (0%)	2 (6.9%)	2 (28.6%)	0.01
STSC	28 (96.6%)	28 (96.6%)	1 (14.3%)	<0.001

*P*-value for One-way analysis of variance, or Chi-square test (comparing three groups).

NICU, Neonatal Intensive Care Unit.

STSC, Skin to Skin Contact.

As for delivery outcomes affecting OXT levels, the association between duration of labor (min), instrumental delivery, induction of labor, augmentation of labor and oxytocin was analyzed, but these were not associated.

### 3.3 Postpartum outcomes

The postpartum outcomes of the participants are shown in Table 3. There were significant differences in Time to walk after childbirth (p < 0.01), Frequency of feeding (0–9 o'clock at 1 day) (Breastfeeding p < 0.04, Expression of milk p < 0.009, Formula p < 0.001), Frequency of feeding (0–9 o'clock at 5 days) (Breastfeeding p < 0.02, Expression of milk p < 0.33, Formula p < 0.009), and changes in fatigue score between 1 day and 5 days except for subjective symptoms of fatigue at 1 day and 5 days (p < 0.01), and changes in maternity blues score between 1 day and 5 days (p < 0.07). Multiple comparisons showed that women with emergency CS had significantly longer walking times than women with EA and without EA (p < 0.001, respectively). The frequency of expression milk and formula was significantly higher at 1 day for women with emergency CS than for women with EA and without EA (expression milk: with EA p = 0.006, without EA p = 0.01; formula: with EA p = 0.01, without EA p < 0.001). The frequency of milk at 5 days was significantly higher for women with EA than for women without EA (p = 0.04). The frequency of breastfeeding was lower for women with EA than for women without EA (p = 0.04).

**Table 3:** Postpartum outcomes (N = 65).

	With epidural anesthesia (n = 29)	Without epidural anesthesia (n = 29)	Emergency cesarean section (n = 7)	*P*-value
***Postpartum***				
Time to walk after childbirth	469.4 (SD363.3)	244.3 (SD245.2)		0.01
Frequency of feeding (0 to 9 o'clock at 1 day)				
Breastfeeding	2.0 (SD0.7)	2.6 (SD2.0)	0.7 (SD1.8)	0.04
Expression of milk	0.03 (SD0.1)	0.14 (SD0.5)	0.8 (SD1.4)	0.009
Fomula	1.76 (SD1.0)	1.3 (SD1.1)	3.0 (SD0.5)	0.001
Breastfeeding				
Frequency of feeding (0 to 9 o'clock at 5 days)				
Breastfeeding	3.2 (SD1.4)	4.1 (SD1.3)	2.8 (SD1.7)	0.02
Expression of milk	0.6 (SD1.0)	0.5 (SD1.1)	1.29 (SD1.2)	0.33
Fomula	2.4 (SD1.4)	1.3 (SD1.3)	2.43 (SD1.2)	0.009
***Subjective symptoms of fatigue***				
Subjective symptoms of fatigue at 1 day (5–25) ∗(n = 45)				
Ⅰ: Feeling of drowsiness	12.7 (SD4.68)	12.5 (SD3.73)	12.0 (SD5.47)	0.93
Ⅱ: Feeling of instability	7.26 (SD7.17)	7.63 (SD2.90)	8.60 (SD3.64)	0.59
Ⅲ: Feeling of uneasiness	8.43 (SD3.20)	7.13 (SD2.45)	6.40 (SD2.07)	0.18
Ⅳ: Feeling of local pain or dullness	10.3 (SD3.62)	10.8 (SD4.42)	9.60 (SD3.36)	0.80
Ⅴ: Feeling of eyestrains	7.30 (SD3.54)	6.68 (SD2.74)	6.40 (SD3.13)	0.14
Subjective symptoms of fatigue at 5 days (5–25)∗(n = 45)				
Ⅰ: Feeling of drowsiness	16.7 (SD2.74)	14.2 (SD4.72)	14.6 (SD4.03)	0.98
Ⅱ: Feeling of instability	7.91 (SD2.53)	7.09 (SD2.28)	7.20 (SD2.16)	0.50
Ⅲ: Feeling of uneasiness	7.78 (SD3.24)	7.18 (SD2.44)	9.80 (SD2.86)	0.19
Ⅳ: Feeling of local pain or dullness	13.0 (SD4.54)	12.8 (SD4.80)	13.2 (SD3.63)	0.97
Ⅴ: Feeling of eyestrains	7.43 (SD3.52)	8.36 (SD4.27)	9.80 (SD4.02)	0.61
***Changes in fatigue score between 1 day and 5 days***	14.8 (SD19.7)	0.31 (SD20.7)	4.00 (SD18.5)	0.01
***Changes in maternity blues score between 1 day and 5 days***	2.96 (SD3.65)	1.20 (SD2.39)	1.00 (SD3.51)	0.07

*P*-value for One-way analysis of variance.

There was no significant difference in the number of women diagnosed with maternity blues at 1 day and 5 days after childbirth in the three groups (1 day: p = 0.66, 5 days: p = 0.50). The fatigue scores (VAS: 0–100 mm) from 1 day to 5 days after childbirth were as follows: for women with EA, 50.7 (SD20.2) to 65.5 (SD21.3); for women without EA, 45.7 (SD22.2) to 45.6 (SD24.6); for women with emergency CS 43.8 (SD14.3) to 48.2 (SD13.0). There was a significant difference in the fatigue scores at 5 days after childbirth among the three groups (p = 0.004). Multiple comparisons showed that women with EA had significantly higher fatigue scores than women without EA (p = 0.003).

Similarly, changes in the fatigue scores between 1 day and 5 days showed that women with EA had increased fatigue compared with women without EA (p = 0.02).

### 3.4 Changes in salivary OXT levels of women with emergency CS and changes in OXT levels in three groups

**Fig. 1:**
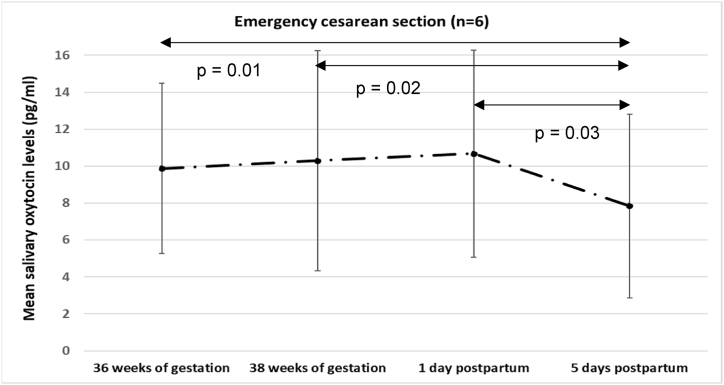
Changes in salivary OXT levels of women with emergency cesarean section.

The mean salivary OXT levels of women with emergency CS was significantly lower at 5 days after childbirth than at 36 weeks of gestation, 38 weeks of gestation, and 1 day after childbirth (36 weeks of gestation: p = 0.01; 38 weeks of gestation: p = 0.02; 1 day after childbirth: p = 0.03) (Fig. 1).

**Fig. 2:**
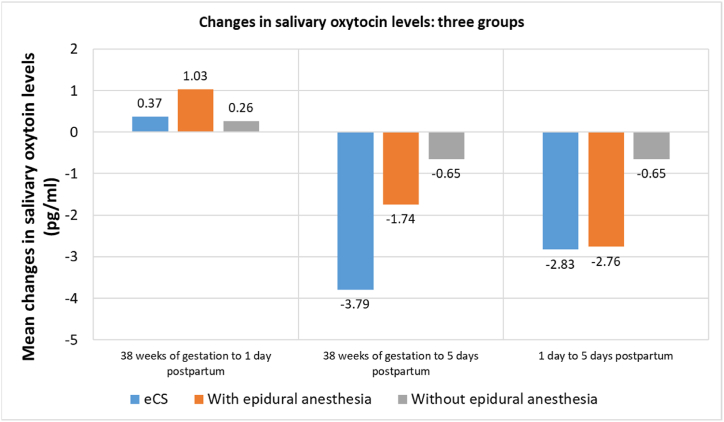
Changes in OXY levels in three groups.

There was no significant difference in mode of delivery and changes in mean OXT levels (38 weeks–1 day after childbirth p = 0.66; 38 weeks to 5 days after childbirth: p = 0.19; 1 day to 5 days after childbirth: p = 0.11) (Fig. 2).

### 3.5 Correlation with changes in mean salivary OXT levels and changes in maternity blues score/changes in fatigue scores between 1 day and 5 days after childbirth

**Fig. 3:**
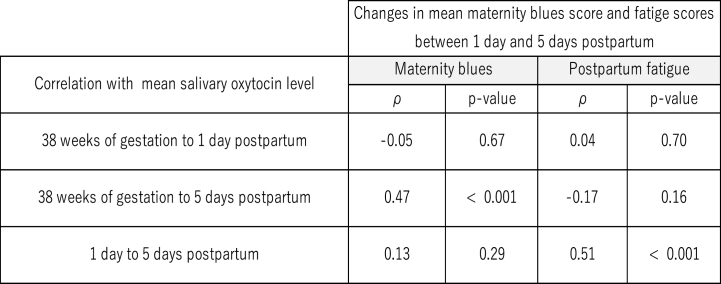
Correlation with changes in mean salivary OXY levels and changes in maternity blues score/changes in fatigue score between 1 day and 5 days postpartum.

There was a significant correlation between changes in the mean maternity blues scores between 1 day and 5 days after childbirth, and the mean changes in OXT levels from 38 weeks gestation to 5 days after childbirth (N = 65, ρ = 0.47, p < 0.001) (Fig. 3). There was a significant correlation between changes in the mean fatigue scores between 1 day and 5 days after childbirth, and the mean changes in salivary OXT levels between 1 day and 5 days after childbirth (ρ = 0.51, p < 0.001).

By mode of delivery, the change in salivary OXT levels was ρ = 0.49, p = 0.003 for women with EA (n = 29) from 38 weeks gestation to 5 days after childbirth, and a larger change in salivary OXT levels was associated with a larger change in maternity blues score between 1 day and 5 days. Women without EA (n = 29) were negatively correlated (ρ = −0.61, p < 0.001). The changes in maternity blues score and OXT levels from 1 day to 5 days after childbirth were weakly correlated, although not significantly for women with emergency CS (n = 6, ρ = 0.24, p = 0.64).

The changes in the fatigue scores and OXT levels from 1 day to 5 days after childbirth were weakly correlated only for women with EA (n = 29, ρ = 0.27, p = 0.13).

## 4 Discussion

### 4.1 Changes in salivary OXT levels in women with emergency CS

To the best of our knowledge, this was the first study to measure the salivary OXT levels in women with emergency CS.

The OXT levels in women with emergency CS increased from late pregnancy to 1 day after childbirth and decreased significantly through 5 days after childbirth. Particularly, there was a significant decrease in the mean salivary OXT levels at 5 days after childbirth compared with at 1 day after childbirth (p = 0.03).

The present results are supported by findings of previous studies. As for the number of OXT receptors in the uterus, it has been reported that it peaks around 39 weeks of gestation [21]. A study by De Geest et al. (1985) [22] showed that plasma OXT levels increased significantly with advancing weeks of gestation. Prevost et al. (2014) [23] reported that the rate of plasma OXT elevation from early to late pregnancy was 73.2%. Uvnäs-Moberg et al. (2019) [24] reported that the OXT levels increases 3–4 times from late pregnancy to delivery. Stock et al. (1991) [25] reported a gradual increase in the plasma OXT levels when approaching delivery, and a decrease in the plasma OXT levels until 8 weeks after delivery. The salivary OXT levels was elevated at 38–39 weeks of gestation, consistent with the studies [[22], [23], [24], [25]].

In the present study, the OXT levels peaked at 1–2 days after childbirth. Although this result was not consistent with that of Stock et al. (1991) [25], it was consistent with those of Shishido et al. (2021) [13] and Jobst et al. (2016) [26]. Jobst et al. (2016) [26] measured the plasma OXT levels at 35 and 38 weeks of gestation, within 2 days, at 7 weeks, and 6 months after childbirth in 100 low-risk pregnant women. They found that plasma OXT levels increased from 35 weeks of pregnancy up to 2 days after childbirth in mothers without depressive symptoms (n = 60).

The changes in the salivary OXT levels in women with emergency CS were similar to those in women with EA. The change in the salivary OXT levels from late pregnancy to 5 days after childbirth was also the lowest in women with emergency CS. For women with EA, the decrease in the OXT levels indicated an effect on postpartum fatigue. However, the postpartum fatigue of women who had an emergency CS recovered from 1 day to 5 days after childbirth [13].

Some factors affecting the lower salivary OXT levels from 1 day to 5 days after childbirth in women with emergency CS may include an increase in exogenous OXT units, fewer performed STSC, inability to share the room with the mother and child owing to a delay in first walking, low frequency of breastfeeding after CS, and low breastfeeding frequency at 5 days after childbirth. In their systematic review, Scatliffe et al. (2019) [27] reported that an increased maternal OXT levels has a significant positive correlation to more affectionate contact behaviors in mothers following the mother and infant contact, synchrony, and engagement. Whitle et al. (2020) [28] reported that breastfeeding women had higher mean OXT levels than women who gave formula. In the case of women with emergency CS, their breastfeeding initiation occurs later and they have an increased rate (41%) of breastfeeding difficulties compared with women with planned CS [29]. In their review of breastfeeding, Krol & Grossmann (2018) [30] described that mothers who give breastfeeding benefit from the actions of OXT which helps to significantly reduce physiological and subjective stress, promoting positive emotions and parenting. The effects OXT on the infant are also indicated.

In the present study, it was not possible to analyze this association because of the small sample size; however, it is possible to clarify predictors as the sample size accumulates in the future. In their recent study (2021), Shishido et al. [13] suggested that women without EA had a higher rate of mother-baby rooming-in and a higher frequency of exclusive breastfeeding, which caused fewer changes in salivary OXT levels after childbirth. Therefore, as a midwifery care, more lactation support including nipple stimulations is suggested for women with CS immediately after childbirth.

### 4.2 Association between changes in salivary OXT levels and changes in maternity blues scores

In the present study, there was a moderate correlation between the changes in salivary OXT levels from 38 weeks of gestation to 5 days after childbirth and the changes in maternity blues scores. Regarding mode of delivery, women with EA (ρ = 0.49) and women with emergency CS (ρ = 0.24) were associated with changes in maternity blues.

The more pronounced the decrease in the changes in salivary oxytocin levels, the higher the significance of the maternity blues score. The association between endogenous oxytocin and maternity blues in this study suggests that measuring the changes in oxytocin level from pregnancy to the early postpartum may be useful for early prevention of maternity blues.

It has been reported that the transition from maternity blues to postpartum depression should be prevented [31], and the changes in oxytocin levels could be used as a predictor.

In previous studies, women who have emergency CS report increased stress, sleeplessness, and worry, particularly among primiparous woman [32]. In their network meta-analysis, Sun et al. (2021) [33] reported that women with emergency CS have a significantly increased risk of postpartum depression compared with women with vaginal delivery (43 studies, OR:1.53 [95%CI 1.22 to 1.91]). Luciano et al. (2021) [34] reported that women with maternity blues had an increased risk of increased Edinburgh Postnatal Depression Scale score (OR: 7.79, 95%CI: 6.88 to 8.70). Gerli et al. (2021) [14] also reported that CS was a predictor of maternity blues. Thus, it is estimated that women with emergency CS are more likely to have maternity blues than women with vaginal deliveries or planned CS.

It is necessary to consider the effects of anesthesia and exogenous OXT usage as a basis for the understanding the association between EA and emergency CS. These delivery methods have been reported to significantly increase the use of exogenous OXT. Mikuš et al. (2021) [35] found a significant weak positive correlation between the use of exogenous OXT and the maternity blues score at 3 days postpartum (r = 0.308, p < 0.01).

Taken together, it could be assumed that women with emergency CS may be affected psychologically by the unplanned method of delivery. Importantly, the effects of exogenous OXT must be further examined in future research.

### 4.3 Strength of this study

There are no studies on the association between oxytocin levels and maternity blues that may occur during the early postpartum period.

A strength of this study is that saliva samples were collected at four time points from late pregnancy to early postpartum. Data were also collected continuously postpartum for women who had an emergency CS; therefore, we were able to determine that oxytocin changes in women who had received an EA and women who underwent an emergency CS are very similar. Our knowledge of changes in oxytocin led us to consider the possibility of delayed initiation of child care and lactation immediately postpartum.

### 4.4 Limitations

As the present study used a prospective design primarily involving women who had planned to have vaginal deliveries, the sample of women who had emergency CS was small. Although difficult, collecting data only from women who had emergency CS was a valuable practice. The statistical power was 59% after our study. Previous studies have reported that women with emergency CS had mental health issues such as Post Traumatic Stress Disorder, anxiety, and depression [36]. Therefore, our main goals in subsequent studies are to continue increasing the sample size and to investigate their characteristics in more detail.

This study included data for oxytocin levels with %CV > 10. The data for %CV within 10% are considered more reliable, so future studies should be selective with the data they use for analysis.

There was also a lack of information on basic data and pain scores affecting oxytocin levels with regard to the data collected in this study. Future studies should consider obtaining information on gynaecological diseases, assisted reproductive technologies, labor pain, breast pain, perineal pain, timing of EA, and pain control.

## 5 Conclusion

The changes in OXT levels from 38 weeks of gestation to 5 days after childbirth was found to have a middle association with maternity blues. It could be assumed that women with emergency CS may be affected psychologically by the unplanned method of delivery. Therefore, this study suggests that it is necessary to investigate the mechanism of OXT and maternity blues.

## Author contribution statement

Eri Shishido: Conceived and designed the experiments; Performed the experiments; Analyzed and interpreted the data; Contributed reagents, materials, analysis tools or data; Wrote the paper. Shigeko Horiuchi: Conceived and designed the experiments; Contributed reagents, materials, analysis tools or data; Wrote the paper.

## Data availability statement

Data will be made available on request.

## Additional information

No additional information is available for this paper.

## Declaration of competing interest

The authors declare that they have no known competing financial interests or personal relationships that could have appeared to influence the work reported in this paper.
